# Exploring the aerobic stability, antioxidant and microbial community of *Broussonetia papyrifera* ensiled with ferulic acid esterase-producing *Lactiplantibacillus plantarum* in combination with cellulase and/or xylanase

**DOI:** 10.1186/s12866-025-04185-z

**Published:** 2025-08-23

**Authors:** YiXi Long, Mengxin Li, Ya Su, Qiang Yu, Yuanjiang Rong, Yulong Xi, Hong Sun, Yixiao Xie, Jun Hao, Chao Chen, Yulong Zheng, Fuyu Yang

**Affiliations:** 1 College of Animal Science, Guizhou University, Guiyang, Guizhou 550025 China; 2 College of Animal Science, China Agricultural University, Beijing, 100193 China

**Keywords:** Ferulic acid esterase, Cellulase, *Broussonetia papyrifera* ensiling, Aerobic stability, Microbial community

## Abstract

**Background:**

Ferulic acid possesses certain antioxidant and antibacterial properties. Additionally, ferulic acid esterase (FAE) and cellulolytic enzymes have been associated with synergistic degradation of ferulic acid ester bonds, thereby facilitating greater release of ferulic acid from lignocellulose, which could have important effects on silage quality and aerobic stability.

**Methods:**

This study examined the effects of ensiling *Broussonetia papyrifera* with FAE-producing *Lactiplantibacillus plantarum* (LP), cellulase (CE) and xylanase (XY) under aerobic exposure conditions. The following treatments were used: distilled water (CK), LP, LP + CE, LP + XY and LP + XY + CE. After 60 days of silage treatment, the samples were unsealed for aerobic exposure for 1, 3, 5, or 7 days.

**Results:**

Compared with the CK treatment, the addition of FAE-producing *L. plantarum* significantly (*P* < 0.05) led to lower pH, reduced dry matter loss of the silage and increased lactic acid (LA) concentration after 60 d of ensiling (especially for the LP + CE and LP + CE + XY groups). During the aerobic exposure stage, the combined treatment with LP and enzymes effectively inhibited the increase in pH, significantly reduced the rate of dry matter loss and increased the LA concentration and aerobic stability of the silage (*P* < 0.05). Moreover, the LP + CE and LP + CE + XY treatment groups exhibited higher ferulic acid levels than the other groups did, corresponding with greater aerobic stability, especially for the LP + CE group, which remained stable. In this group, the pH values showed minimal change, increasing by only 0.31 (4.24–4.55) after 7 days of aerobic exposure. In addition, the LP and enzyme co-treatment was linked to shifts in the microbial community of the silage during aerobic exposure, with increased relative abundance of *Lactiplantibacillus plantarum*, and its abundance positively correlated with lactic acid and ferulic acid concentrations, while negatively correlated with ammonia nitrogen; and inhibited proliferation of spoilage-related bacteria (*Enterobacter*,* Gluconobacter* and *Cladosporium*).

**Conclusions:**

The combination of FAE-producing *L. plantarum* and cellulase can be used as an effective method to increase the preservation efficiency and aerobic stability of *B. papyrifera* silage.

**Graphical Abstract:**

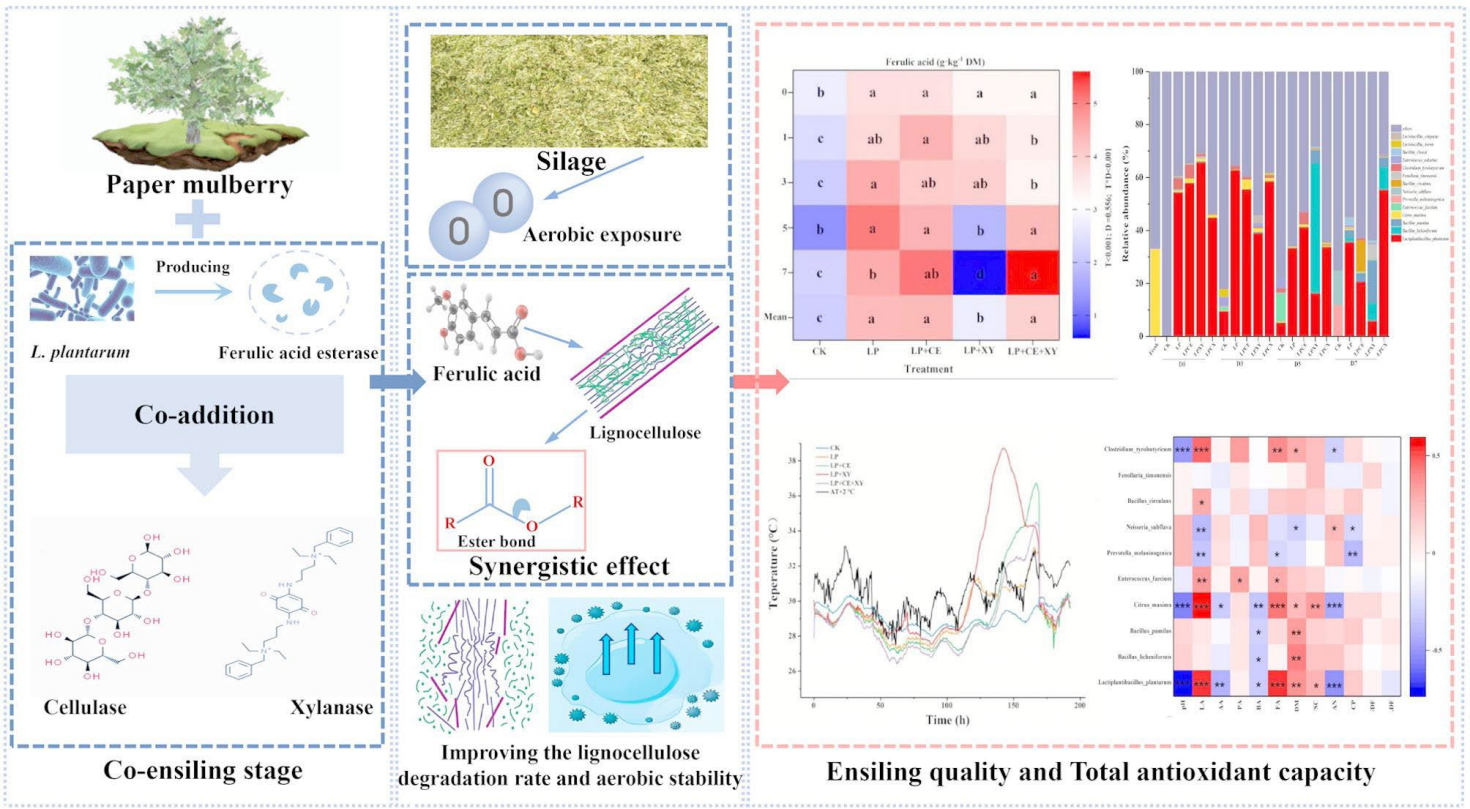

## Background

Paper mulberry (*Broussonetia papyrifera*) is a perennial tree belonging to the Moraceae family that is fast-growing and highly adaptable and has a high yield and nutritional value [1, 2]. Therefore, it can be utilized as protein feed for livestock. Ensiling is a traditional method for ensuring a year-round supply and improving the storage efficiency of paper mulberry, especially in southwestern China [3, 4]. Several studies have focused on additive silage of *B. papyrifera* to solve the problems of low amounts of epiphytic lactic acid bacteria (LAB), a high buffering capacity and a high fibre content [1, 5]. Our previous study suggested that adding ferulic acid esterase (FAE)-producing *Lactiplantibacillus plantarum*, cellulase (CE) and xylanase (XY) contribute synergistically to decreasing the *B. papyrifera* silage pH and increasing lignocellulose degradation, lactic acid (LA) content and water-soluble carbohydrate (WSC) levels [5]. However, previous studies have indicated that relatively high contents of residual WSCs and LA provide sufficient substrates for undesirable microorganisms and accelerate aerobic deterioration [6, 7]. Therefore, the effects of adding FAE-producing *L. plantarum*, CE and XY on the aerobic stability of *B. papyrifera* silage need to be explored.

The cross-linking of lignin‒carbohydrate complexes through bonds such as ferulic acid ester bonds limits lignocellulose degradation and restricts the release of ferulic acid (FA) from plant cell walls [8–10]. Previous studies have shown that FA has potent antioxidant and antimicrobial properties [11, 12]. The antibacterial effect of ferulic acid is consistent with the results that the polyphenolic compounds have a strong inhibitory effect on Gram-positive bacteria [13]. It has a strong scavenging effect on three types of free radicals: hydroxyl radicals, superoxide anions, and DPPH. The antibacterial mechanism is related to interfering with protein synthesis and destroying the permeability of the cell wall and cell membrane [14]. Furthermore, synergistic interactions between ferulic acid esterase (FAE) and xylanase (XY) have been associated with increased FA release from substrates. For example, combining FAE and XY led to increased FA yields by tenfold compared with those of FAE alone in wheat bran [15]. However, few studies have focused on the release of FA from silage through the addition of FAE-producing *L. plantarum*, CE and XY and its effects on silage quality and aerobic stability.

In tropical and subtropical regions, silage spoilage is prevalent due to high temperatures and humidity, particularly during aerobic exposure, which promotes microbial proliferation, an elevated pH, and nutrient loss [16]. A previous study revealed that the addition of *Lactiplantibacillus brevis* improved the aerobic stability of paper mulberry silage, but the addition of *L. plantarum* had no effect or even a negative effect on aerobic stability [17, 18]. In addition, *B. papyrifera* has antioxidant, antibacterial and anti-inflammatory activities because it contains more than 40 polyphenols and polyflavonoids [19]. Moreover, mulberry ensiled with LAB and cellulase additives can improve the antioxidant activity of materials [20]. Consequently, we hypothesize that supplementing *L. plantarum*, CE, and XY may optimize FA release and preserve the antioxidant properties of *B. papyrifera* silage.

Therefore, in this study, FAE-producing lactic acid bacteria in combination with CE and/or XY were added to *B. papyrifera* silage to explore the fermentation characteristics, antioxidants and microbial communities of the silages during aerobic exposure.

## Materials and methods

### Raw silage materials and silage Preparation

The first-cut paper mulberry was manually harvested at a height of 1.2 m from Changshun County, Guizhou Province, China, in May 2023. The raw materials were wilted until the moisture content reached approximately 66% and then cut into 1–2 cm pieces using a chopper. Keep a portion of fresh raw material at 4°C for microbial counting by the spread plate method on the following day, freeze a portion of fresh raw material at −20°C for later analysis. The FAE-producing strain of *L. plantarum* was isolated from rumen fluid. Detailed information can be found in our previous study [10]. Cellulase and xylanase are commercial enzymes with activities of 50 U mg^−1^ and 20 U mg^−1^, respectively.

The chopped raw material was treated with distilled water (CK), FAE-producing *L. plantarum* (LP), LP + CE, LP + XY or LP + XY + CE. Lactic acid bacteria (LAB) were inoculated at a concentration of 1 × 10^6^ colony forming units (cfus) g^−1^ fresh matter (FM). The amount of degrading enzymes applied to the LP + CE and LP + XY groups was 50 U g^−1^ FM, and the LP + XY + CE group was treated with 25 U g^−1^ FM CE and 25 U g^−1^ FM XY. The above additives were mixed evenly with the chopped materials and randomly loaded into silage bags. Each bag was filled with approximately 700 g of sample and then vacuum sealed. Each treatment was repeated 15 times, and the samples were placed in a dark room at room temperature (27 ± 1°C). After 60 days of ensiling (0 day of aerobic exposure), 3 bags per treatment group were randomly sampled for analysis of the chemical composition, fermentation characteristics and microbial communities.

### Aerobic stability analysis

The silages were subjected to a 7-day aerobic stability test using the method described by Bai et al. [21]. Briefly, the silage bags were opened and loosened on day 60, and then the materials were placed in 2 L polyethylene bottles and covered with two layers of medical gauze to prevent the surface of the samples from mutual contamination and debris from falling. The bottle was placed in the dark (27 ± 1°C), and the temperature of each bottle was recorded every 30 min using an automatic temperature recorder (TOPRIE, TP9000, Shenzhen Topurui Electronics Co. Ltd.). The aerobic stability time was defined as the time until the sample temperature exceeded the ambient temperature by 2℃ [22]. The nutritional characteristics, fermentation quality and microbial community were measured by sampling after 1, 3, 5, and 7 days of aerobic exposure.

### Fermentation characteristics and chemical component analyses

After 60 days of ensiling and 1, 3, 5, and 7 days of aerobic exposure, 20 g of silage samples were thoroughly mixed with 180 mL of sterile distilled water and filtered through 4 layers of gauze [23]. The pH values of the extracts were immediately determined using a pH meter (PHS-3E, Shanghai INESA Scientific Instruments Co., Ltd., Shanghai, China). Remaining portions of the extracts were stored at −80℃ to analyse the ammonia nitrogen (NH_3_-N) and organic acid concentrations. The NH_3_-N content was determined using the method described by Broderick and Kang (1980). The analysis of lactic acid (LA), acetic acid (AA), propionic acid (PA) and butyric acid (BA) concentrations was conducted with a high-performance liquid chromatography system (LC-20 A, Shimadzu, Tokyo, Japan), as described by Xia et al. [24]. The FA content was analysed using an HPLC instrument (LC-20 A, Shimadzu, Tokyo, Japan) equipped with a DIKMA Diamonsil C18 column (4.6 mm × 250 mm; Dikma Technologies, China), following a previously described method [23]. The counts of the LAB and yeast populations were determined by the methods reported by Li et al. [4].

The total antioxidant capacity (T-AOC), glutathione peroxidase (GSH-Px) activity and catalase (CAT) activity were measured using commercial assay kits purchased from Nanjing Jiancheng Bioengineering Institute, Nanjing, China (A015-2-1, A001-3-2, A005-1-2, and A007-1-1, respectively). The samples were incubated in accordance with the manufacturer’s instructions.

The dry matter (DM) of the silage samples was analysed via oven-drying at 65℃ for 48 h. For the chemical composition analysis, the dried samples were ground through a 1 mm screen with a grinder. A Kjeldahl nitrogen analyser (Kjeltec 8400, FOSS, Hoganas, Sweden) was used to measure the crude protein (CP) content. The WSC content was determined using a previously described method [25]. Neutral detergent fibre (aNDF) and acid detergent fibre (ADF) were measured according to the methods of Van Soest et al. [26], and a heat-stable amylase (FAA, Ankom Technology, Macedon, NY) was added when aNDF was measured. The DM loss was calculated with the following formula: DM loss (%) = 1000 × [1-(silage weight at opening/fresh sample weight)].

### Microbial community analysis

Fresh *B. papyrifera* silage, which was ensiled for 60 days and subjected to 3, 5, and 7 days of aerobic exposure, was sampled to extract total microbial DNA using a previously described method (Ye et al., 2011). The total DNA was extracted from the silage samples via the cetyltrimethylammonium bromide (CTAB) method. Specific primers for 16 S rRNA (27 F-GATCCTGGCTCAG; 1492R-GNTACCTTGTTAC GACTT) were selected to construct a clone library of the full-length 16 S rRNA gene. The primer pairs ITS5-1737-F (GGA AGT AAA AGT CGT AAC AAG G) and ITS2-2043R (GCTGCGTTCTTCATCGATGC) were used to amplify the ITS gene. All PCRs were performed with 15 µL of Phusion^®^ High-Fidelity PCR Master Mix (New England Biolabs), 0.2 µM forwards and reverse primers, and approximately 10 ng of template DNA. Thermal cycling included initial denaturation at 98℃ for 1 min, 30 cycles of denaturation at 98℃ for 10 s, annealing at 50℃ for 30 s, and elongation at 72℃ for 30 s, followed by 72℃ for 5 min. The libraries were sequenced on the PacBio Sequel platform. The raw sequences were first processed by Novogene Technology Co., Ltd. (Beijing, China) and subsequently processed on the Magic platform (https://magic.novogene.com/) for alpha diversity, coverage value, PCoA and other analyses.

### Data processing and statistical analysis

The aNDF and ADF degradation rate was calculated according the following equation:

$$\mathrm{aNDF}/\mathrm{ADF}\;\mathrm{degradation}\;\mathrm{rate}\;(\%)=100\times\frac{\mathrm{aNDF}/{\mathrm{ADF}}_{\mathrm{fresh}\;\mathrm{sample}}-\mathrm{aNDF}/{\mathrm{ADF}}_{\mathrm{sialge}}}{\mathrm{aNDF}/{\mathrm{ADF}}_{\mathrm{fresh}\;\mathrm{sample}}}$$

IBM SPSS 23.0 software (SPSS Inc., Chicago, IL, United States) was used to conduct statistical analyses of the data. One-way and two-way analyses of variance (ANOVAs) were performed on the fixed effects of additives and ensiling time. Duncan’s multiple range test was used to separate the means among the treatment groups (differences were considered significant if *P* < 0.05). Heatmaps based on Spearman’s correlation coefficients between the microbial population and silage fermentation parameters were generated using SPSS software.

## Results and discussion

### Characteristic of Raw *B. papyrifera* materials

The characteristic of fresh *B. papyrifera* before ensiling are shown in Table 1. The DM of the raw materials was 335.68 g·kg^−1^ FM, and the aNDF, ADF and CP contents were 474.04, 257.68 and 149.51 g·kg^−1^ DM, respectively, which were higher than the values reported in previous studies [15], possibly because of differences in harvest time and climate. The pH of the raw *B. papyrifera* was 7.14, which was higher than that reported by Zhang et al. [18]. WSC is known as the main substrate for microbial (especially LAB) fermentation during ensiling [27]; in this study, the WSC content was 68.42 g·kg^−1^ DM. The population of epiphytic LAB (6.03 log cfus g^−1^ FM) on *B. papyrifera* was lower than that of yeasts (6.14 log cfus g^−1^ FM). A previous study indicated that *B. papyrifera* has antioxidant properties. In this research, the activities of T-AOC, LOX and GSH-Px were 100.91, 3196.67 and 452.79 U·g^−1^ FW, respectively.

**Table 1:** Characteristic of fresh paper mulberry at harvest (*n* = 3)

Items	Mean ± standard deviation
Dry matter (g·kg^−1^ FM)	335.68 ± 16.48
pH	7.14 ± 0.06
Neutral detergent fiber (g·kg^−1^ DM)	474.04 ± 23.67
Acid detergent fiber (g·kg^−1^ DM)	257.68 ± 8.23
Crude protein (g·kg^−1^ DM)	149.51 ± 5.89
WSC (g·kg^−1^ DM)	68.42 ± 0.32
LAB (log cfu g^−1^ FM)	6.03 ± 0.12
Yeast (log cfu g^−^1 FM)	6.14 ± 0.11
T-AOC (U·g^−1^ FM)	100.91 ± 6.13
LOX (U·g^−1^ FM)	3196.67 ± 418.74
GSH-Px (U·g^−1^ FM)	452.79 ± 30.41

*FM* Fresh matter, *DM* Dry matter, *WSC* Water soluble carbohydrates, *LAB* Lactic bacteria, *T-AOC* Total antioxidant capacity, *LOX* Lipoxygenase, *GSH-Px* Glutathione peroxidase

### pH value and temperature of *B. papyrifera* silages during the aerobic exposure

**Fig. 1:**
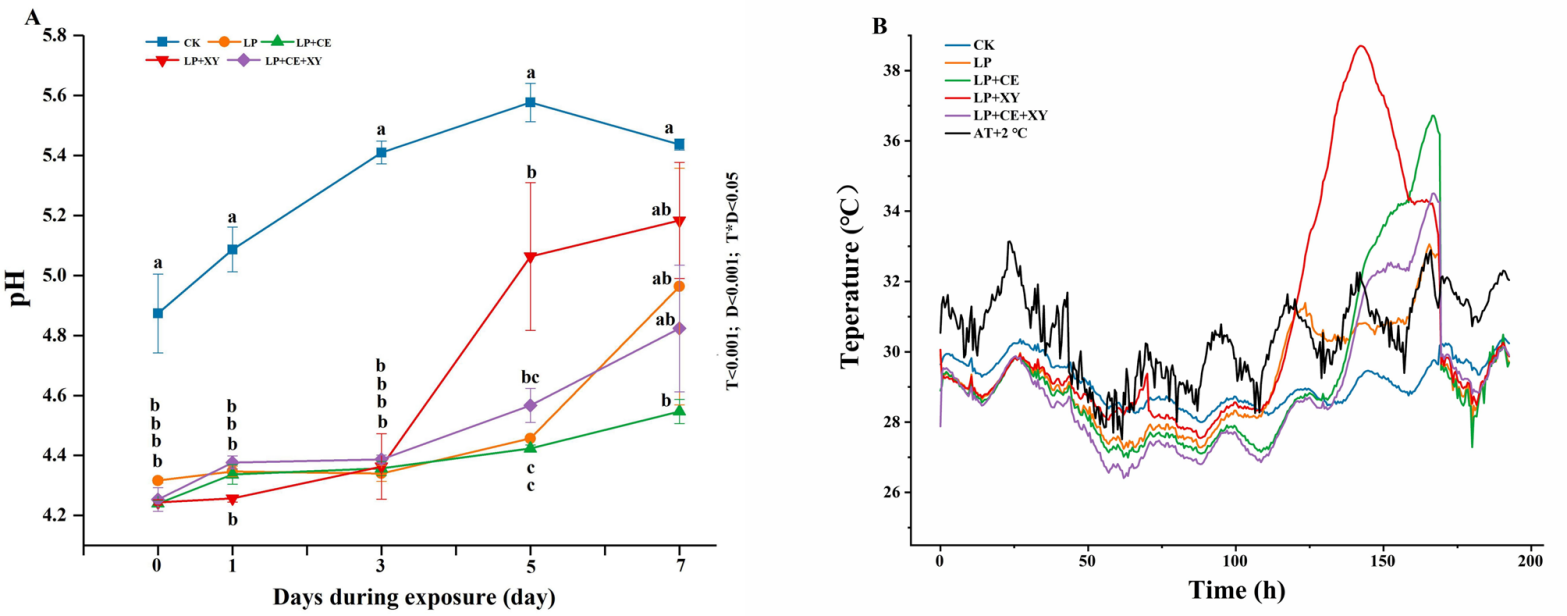
The effect of different treatment on the pH **A** and temperature **B** dynamics of *Broussonetia papyrifera* silage during the aerobic exposure. LP, *L. plantarum*; LP + CE, *L. plantarum* + cellulase; LP + XY, *L. plantarum* + xylanase; LP + CE + XY, *L. plantarum* + cellulase + xylanase; AT, ambient temperature + 2℃. D, Ensiling day; T, additive; D*T, interaction between ensiling day and additive

Changes in pH and temperature of the silage sample during aerobic exposure are presented in Fig. 1. The additive × ensiling day (T × D) interaction affected pH (*P* < 0.05), and T and D significantly influenced pH (*P* < 0.001).

A change in the silage pH is considered a vital indicator that affects microbial activity, silage quality and aerobic stability. In the present study, after 60 days of ensiling (0 d of aerobic exposure), the addition of FAE-LAB significantly decreased the silage pH compared with that of the CK treatment (*P* < 0.05), especially in the bacterial–enzyme treatment groups (LP + CE, LP + XY, and LP + CE + XY), similar to our previous findings [10]. The pH of the LP treatment group increased slightly during the first 3 days of aerobic exposure; subsequently, a substantial increase in the pH of the LP + XY, LP and LP + CE + XY groups was observed after 5, 7 and 7 days of aerobic exposure, respectively. This phenomenon was attributed to the multiplication of aerobic microorganisms, which led to increased pH and nutrient loss [7, 16]. A previous study indicated that an increase in pH could be a measure of aerobic stability and that a pH increase greater than 0.5 compared with the initial value is considered aerobic instability [16]. Therefore, the CK-treated silages were first subjected to aerobic spoilage after 3 d of aerobic exposure, followed by the LP + XY groups (5 days), LP (7 days) and LP + CE + XY groups (7 days). The pH of the LP + CE group after 7 d of aerobic exposure was 4.55, which only increased 0.31 from the initial value (4.24), suggesting high aerobic stability. The more rapid aerobic deterioration in the CK and LP + XY silages than in the LP + CE and LP + CE + XY silages could be due to the presence of undesirable bacteria such as *Ralstonia* and *Acetobacter*, which degrade LA and accelerate aerobic deterioration [7].

As shown in Fig. 1B, the CK silages presented an earlier and greater temperature increase than did the other treatment groups, followed by the LP + XY samples. The higher pH of the CK silages promoted the reproduction and metabolism of aerobic and harmful microorganisms, generated a large amount of heat and initiated heating in the silages [28, 29]. According to the standard for aerobic spoilage, silage is consider spoiled when its temperature is more than 2 ℃ above the ambient temperature [22]. The LP + CE and LP + CE + XY silages remained stable (above 140 h) during the period of aerobic exposure, followed by the LP silages (approximately 120 h). However, the LP + XY silages (approximately 50 h) became unstable (approximately 50 h) after 2 d of aerobic exposure. Overall, the changes in silage temperature were similar to the changes in the pH across all groups. WSC is the main energy source of lactic acid bacteria. A high content of WSC will promote the rapid reproduction of lactic acid bacteria, previous studies reported that adding *L. plantarum* resulted in the highest temperature because more LA was produced and that LA could serve as a substrate for lactate-assimilating yeasts, leading to yeast proliferation and heat production [30]. During the period of aerobic exposure, the WSC content and LA content in the CK group were the lowest, this can explain why the temperature did not significantly increase after the control group was exposed to air. The addition of *L. plantarum* to the LP + CE and LP + CE + XY groups increased aerobic stability, possibly because more FA was produced by FAE-producing LAB and has synergistic effects with enzymes, and FA has strong antimicrobial properties [11, 12].

### Fermentation characteristics and antioxidant of *B. papyrifera* silages during the aerobic exposure

**Fig. 2:**
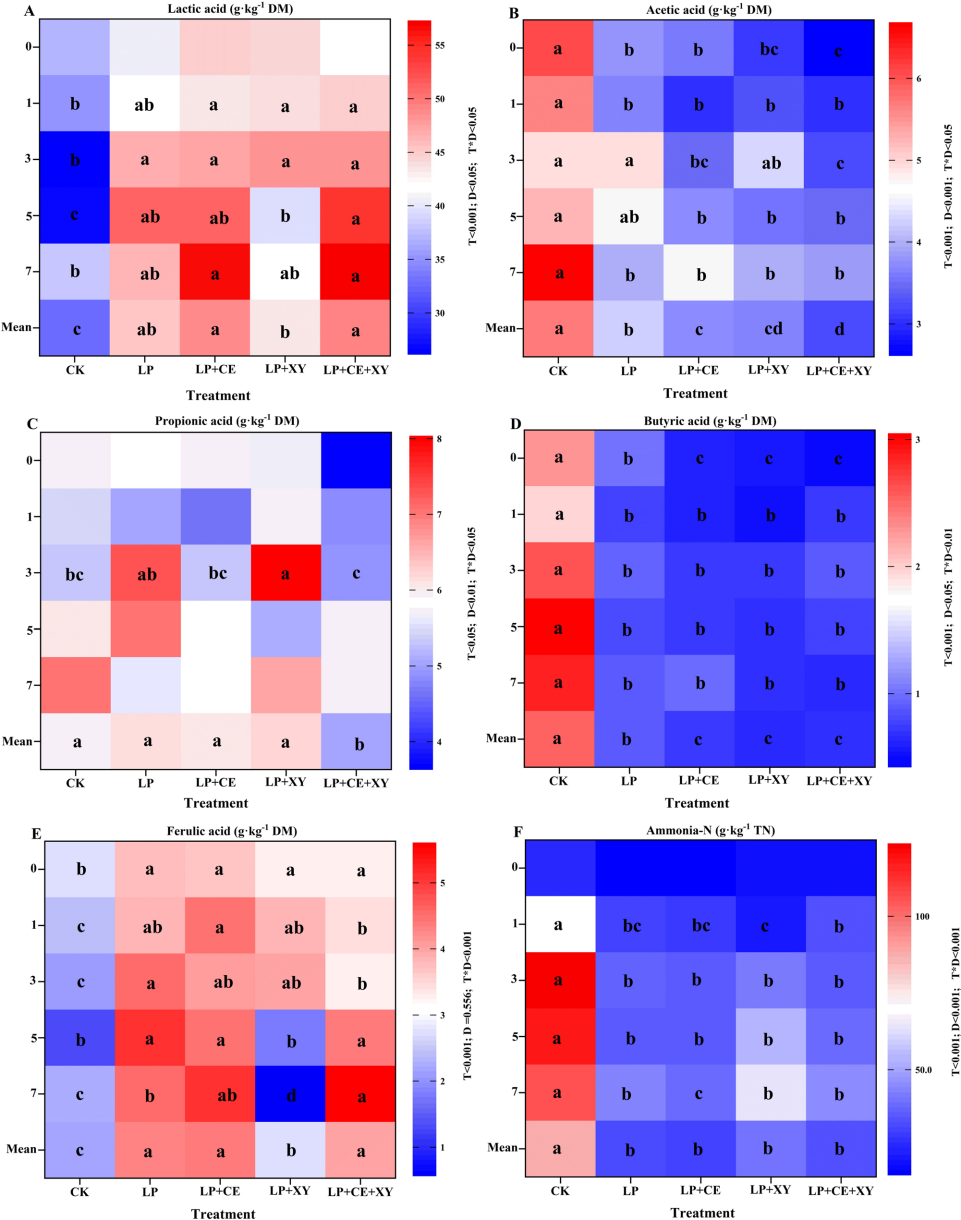
Change of organic acid **A**-**E** and ammonia-N **F** of *Broussonetia papyrifera* silage fermented with different treatment during the aerobic exposure. LP, *L. plantarum*; LP + CE, *L. plantarum* + cellulase; LP + XY, *L. plantarum* + xylanase; LP + CE + XY, *L. plantarum* + cellulase + xylanase. Mean, Treatment mean of the 0, 1, 3, 5, 7 day; D, Ensiling day; T, additive; D*T, interaction between ensiling day and additive. The significant difference (*P* < 0.05) between different treatments on the same days is represented by the different lowercase letters (a-d)

The dynamic changes in the LA, AA, BA, PA, FA and NH_3_-N contents of *B. papyrifera* silages during aerobic exposure are presented in Fig. 2. In the present study, the additive × ensiling day (T × D) interaction affected the LA (*P* < 0.05), AA (*P* < 0.05), PA (*P* < 0.05), BA (*P* < 0.05), FA (*P* < 0.001) and NH_3_-N (*P* < 0.001) contents; T significantly influenced the LA (*P* < 0.001), AA (*P* < 0.001), PA (*P* < 0.05), BA (*P* < 0.001), FA (*P* < 0.001) and NH_3_-N (*P* < 0.001) contents; and D significantly influenced the LA (*P* < 0.005), AA (*P* < 0.001), PA (*P* < 0.01), BA (*P* < 0.05) and NH_3_-N (*P* < 0.001) contents.

Lactic acid is produced by LAB and is the most important substance that decreases the pH of silage; during aerobic exposure, lactic acid can also be utilized by aerobic microorganisms and accelerate aerobic deterioration [6, 31]. As shown in Fig. 2A, the addition of *L. plantarum* and degrading enzymes significantly increased the mean LA level of the silages compared with the CK treatment (*P* < 0.05), especially in the bacterial–enzyme treatment group. However, the LA concentration was lower than that in the previous study [5], which may be attributed to the different harvest times and characteristics of the materials. Compared with that of the initial value (36.95 g·kg⁻¹ DM), the LA concentration of the CK silages decreased sharply at 3 days of aerobic exposure (23.26 g·kg⁻¹ DM), and the LA level of the LP + XY silages started to decrease significantly at 5 days of aerobic exposure (*P* < 0.05), which may be correlated with the consumption of lactate-assimilating yeasts [32]. The decrease in the LA content probably resulted in an increase in pH [15, 28], consistent with the change in pH (Fig. 1A). However, the LA concentration in the LP + CE- and LP + CE + XY-treated silages changed slightly with prolonged aerobic exposure, and was significantly higher than that in the CK groups after 7 days of aerobic exposure, which might be attributed to the high relative abundance of homofermentative LAB (Fig. 7) and strong aerobic stability. As shown in Fig. 2B, the AA concentration was essentially less than 6 g kg^−1^ DM, which was markedly lower than the level of LA, especially in the LP treatment groups. These findings indicated that adding homofermentative LAB inhibited heterolactic fermentation and rapidly produced large amounts of LA using the WSC [10, 33]. Compared with those of the other treatment groups, significantly higher AA concentrations (*P* < 0.05) were observed with the CK group, reaching the highest level of 23.26 g·kg⁻¹ DM, probably because many epiphytic heterofermented LAB were present in the raw material and more AA was produced. Previous studies have shown that both AA and PA have significant antifungal effects and increase the aerobic stability of silage. However, compared with those in the other treatment groups, the aerobic stability of the CK-treated silages was relatively low, which might be related to the FA level.

In addition, the PA concentration ranged from 3.65 to 7.12 g·kg⁻¹ DM, and minimal change was observed in each treatment group during aerobic exposure (Fig. 2C), with no significant differences among the CK, LP, LP + CE, and LP + XY groups. However, the LP + CE + XY treatment group had a significantly lower level of PA than did the other groups, differing from the results reported in a previous study involving the same treatment [5]. This phenomenon could be attributed to the differences in the raw materials of *B. papyrifera.* The content of butyric acid, an undesirable fermentation product, should be less than 10 g kg^−1^ DM in high-quality silages. As shown in Fig. 2D, the BA concentration of the silages (except those in the LP treatment group) increased with increasing aerobic exposure time, which might be due to the conversion of LA and AA by some undesirable microorganisms [4]. Compared with the CK treatment group, the addition of FAE-producing *L. plantarum* resulted in a lower BA concentration, especially in the LP + CE, LP + XY and LP + CE + XY treatment groups, which was consistent with previous results [5]. These results indicate that the use of LP inhibited the production of BA and that FAE producing-*L. plantarum*, cellulase and xylanase exerted a synergistic effect on the inhibition of BA production. Ferulic acid can effectively hydrolyse the ferulic acid ester bonds in lignocellulose.

As shown in Fig. 2E, except for the LP + XY group, the FA contents of the silage treatment groups on the 7th day of aerobic exposure were higher than those on Day 0, and the contents of the LP + CE and LP + CE + XY groups were significantly higher than those of the other treatment groups. The addition of cellulase and FAE-producing LAB can synergistically break the ferulic acid ester bond of the lignin–carbohydrate complex, generating more ferulic acid; moreover, the breaking of the ferulic acid ester bond provides more accessible points for the degradation of cellulose, promoting the degradation of cellulose, providing more substrates for LAB fermentation, promoting the proliferation of FAE-producing LAB, and further promoting the release of ferulic acid. FA has strong antioxidant activity and antibacterial effects and has a strong scavenging effect on hydrogen peroxide [34], superoxide free radicals, light free radicals and nitroso peroxides [35, 36]. Studies by W.Addah et al. [37] have shown that increasing the ferulic acid content can prolong the storage duration of barley silage under aerobic exposure and improve its aerobic stability. Therefore, we speculated that the best total antioxidant capacity detected in the LP + CE and LP + CE + XY groups may be due to the production of more ferulic acid. The content of NH3-N in the CK group increased significantly with increasing aerobic exposure time (*P* < 0.001); this is because an increase in pH value promotes the growth and activity of yeast and molds, these microorganisms will break down proteins to produce NH3-N [38]. At 3 and 5 days, it reached 279.4 g·kg⁻¹ TN and 262.9 g·kg⁻¹ TN, respectively (Fig. 2F). The levels in the LP, LP + CE, LP + XY, and LP + CE + XY groups were not significantly different. The results revealed that the addition of *Lactiplantibacillus plantarum* reduced the production of NH_3_-N [39] and inhibited the excessive decomposition of protein, consistent with previous findings [40].

### Nutrient composition of *B. papyrifera* silages during aerobic exposure

**Fig. 3:**
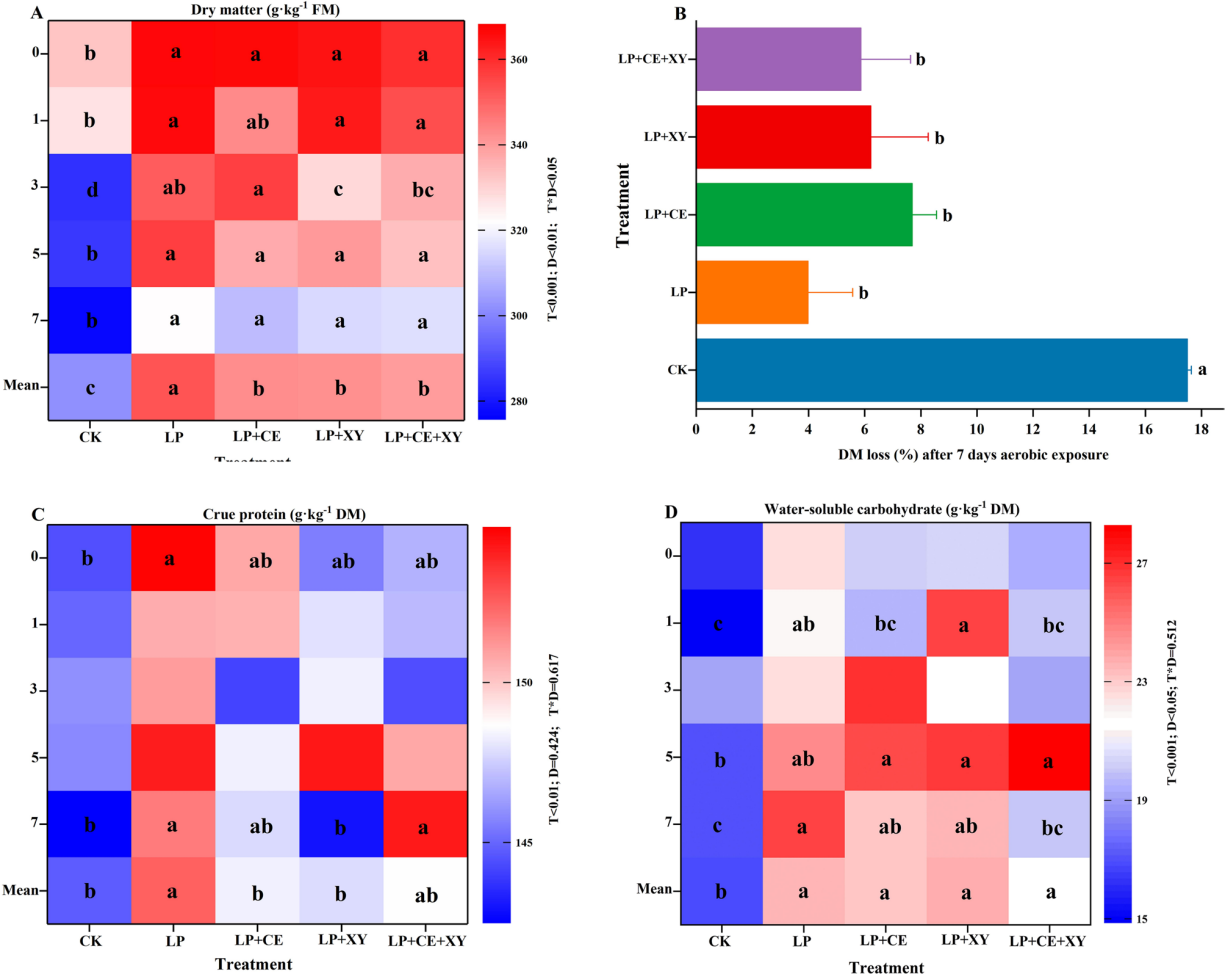
Change of dry matter **A** during the aerobic exposure, DM loss **B** after 7 days aerobic exposure, and change of crude protein **C** and water-soluble carbohydrate **D** of*Broussonetia papyrifera* silage fermented during the aerobic exposure. LP*, L. plantarum*; LP+CE, *L. plantarum*+cellulase; LP+XY, *L. plantarum*+xylanase; LP+CE+XY, *L. plantarum*+cellulase+xylanase. DM, dry matter; Mean, Treatment mean of the 0, 1, 3, 5, 7 day; D, Ensiling day; T, additive; D*T, interaction between ensiling day and additive. The significant difference (*P *< 0.05) between different treatments on the same days is represented by the different lowercase letters (a-d)

As shown in Fig. 3A and B, different additives and different aerobic exposure times had different effects on the DM content and DM loss rate. After 60 days of silage and after 7 days of aerobic exposure, the dry matter content of each treatment group was significantly higher than that of the CK group. The reason for this improvement in the silage DM content may be that rapid acid production inhibited microbial consumption via LAB [41]. The abovementioned results confirmed the synergistic effects among FAE-producing LAB, XY and CE, consistent with previous findings [10]. Compared with that of the raw material, the crude protein content of all the treatment groups in the aerobic exposure stage was significantly reduced (Fig. 3C). According to previous studies, increases in the oxygen content and temperature lead to the colonization of harmful microorganisms and a decrease in the crude protein content [42]. These trends are consistent with those observed in this experiment. After 60 days of silage, the WSC content of each silage was significantly lower than that of the raw material (68.42 g/kg DM), as shown in Fig. 3D. This outcome is because LAB ferment organic acids with WSC as the substrate [43, 44]. Compared with that in the CK group, the WSC content in the combined treatment group was higher because the synergistic effects of LA and enzymes facilitated the degradation of paper mulberry and yielded more WSCs [45].

**Fig. 4:**
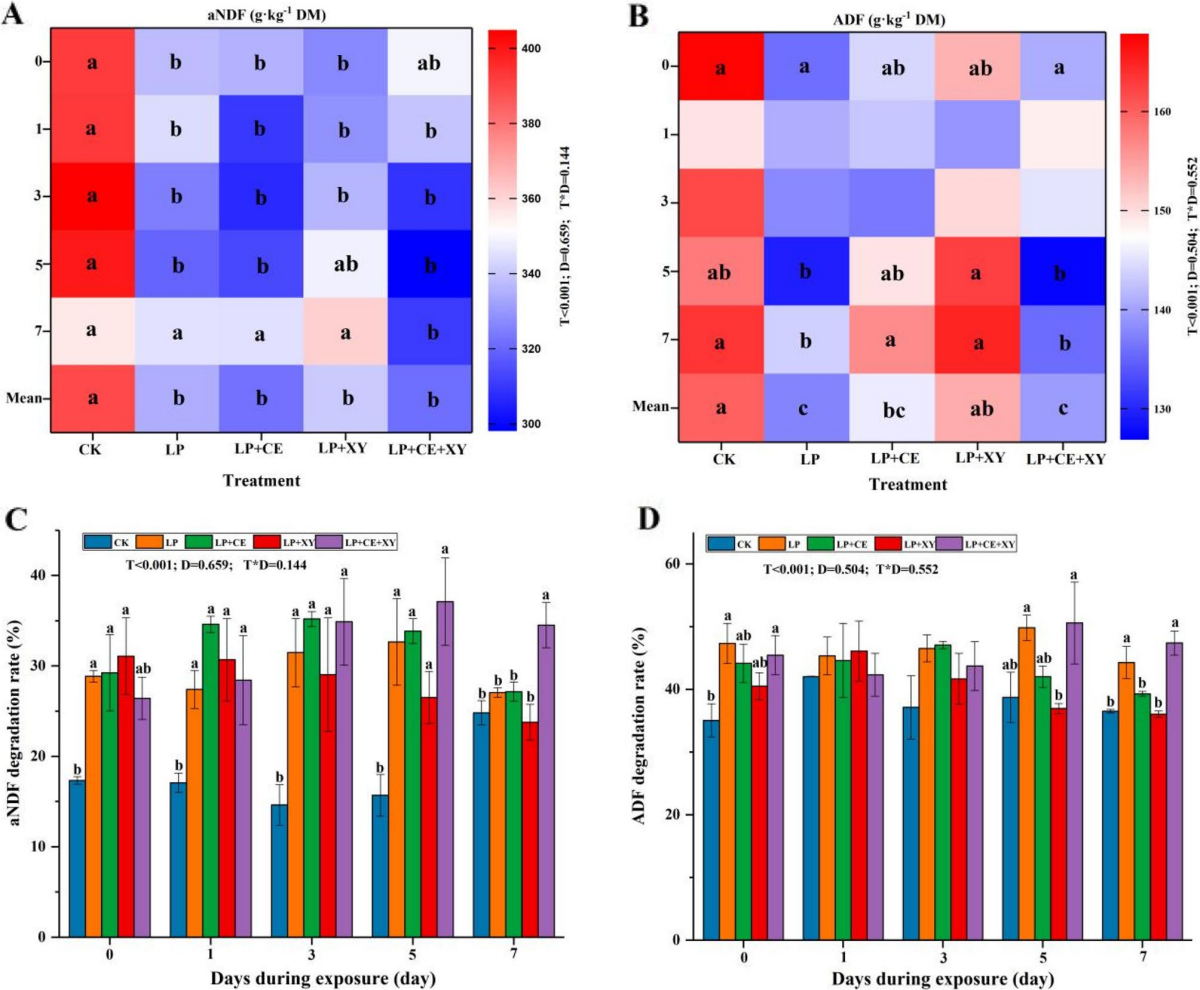
Change of aNDF and ADF **A**-**B** and aNDF and ADF degradation rate **C**-**D** of *Broussonetia papyrifera* silage fermented with different treatment during the aerobic exposure. LP, *L. plantarum*; LP+CE, *L. plantarum*+cellulase; LP+XY, *L. plantarum*+xylanase; LP+CE+XY, *L. plantarum*+cellulase+xylanase. Mean, Treatment mean of the 0, 1, 3, 5, 7 day; D, Ensiling day; T, additive; D*T, interaction between ensiling day and additive. The significant difference (*P *< 0.05) between different treatments on the same days is represented by the different lowercase letters (a-c)

The contents of NDF and ADF in silage were significantly lower than those in raw feed. The NDF contents of the LP + CE group and LP + CE + XY group were lower than those of the LP group and LP + XY group on the 7th day of aerobic exposure (Fig. 4A), which was consistent with the results reported by YANG et al. [44], and the synergistic effect of LP and CE was confirmed again. As shown in Fig. 4B, the ADF contents of the LP, LP + CE, and LP + CE + XY groups were lower than that of the CK group on day 7. Additionally, the extent of NDF and ADF degradation was significantly greater in the LP and LP + CE + XY groups than that of the CK group after 60 days of ensiling and 7 days of aerobic exposure (Fig. 4C and D). These results show that FAE-producing LAB, cellulase and hemicellulase can synergistically degrade lignocellulose, thus increasing the digestibility of forage grass. This finding is also consistent with the conclusion of Lynch J et al. [46]. In the study by Li FH et al. [9], the combined action of FAE-producing LAB and cellulase increased the cellulose conversion efficiency [47].

### Antioxidant activity of *B. papyrifera* silages during aerobic exposure

**Fig. 5:**
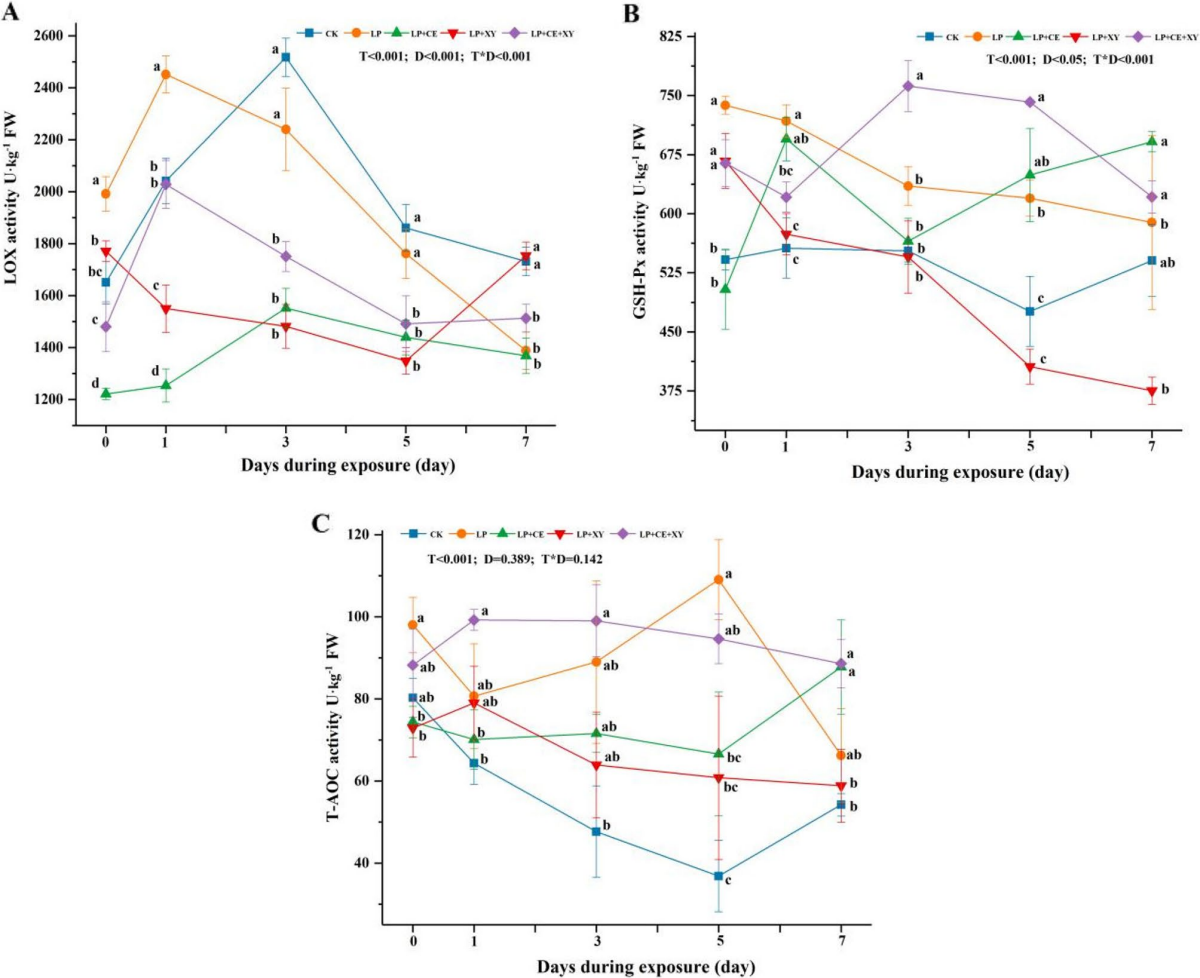
Change of LOX **A**, GSH-Px **B** and T-AOC **C** activity of *Broussonetia papyrifera* silage fermented with different treatment during the aerobic exposure. LP, *L. plantarum*; LP+CE, *L. plantarum*+cellulase; LP+XY, *L. plantarum*+xylanase; LP+CE+XY, *L. plantarum*+cellulase+xylanase. T-AOC, total antioxidant capacity; LOX, Lysyl Oxidase; GSH-Px, glutathione peroxidase. D, Ensiling day; T, additive; D*T, interaction between ensiling day and additive. The significant difference (*P *< 0.05) between different treatments on the same days is represented by the different lowercase letters (a-d)

Lipoxygenase (LOX) is a redox enzyme involved in lipid metabolism and oxidative stress [48]. As shown in Fig. 5A, LOX activity in the LP + CE and LP + CE + XY groups was consistently lower than that in the CK group during aerobic exposure. On day 7 of aerobic exposure, LOX activity was lower in the LP + CE treatment group than in the other treatment groups. These results indicated that the combined treatment with LP and CE effectively controlled the pH and temperature of the feed during the aerobic exposure period, thereby inhibiting the activity of LOX and reducing the oxidation factor of the feed. GSH-Px is a peroxide-reducing enzyme. Except for the LP + CE group, the GSH-Px activity was lower on the seventh day of aerobic exposure than on day 0. In the CK and LP + CE + XY groups, the activity initially rose but subsequently declined within the 7-day aerobic exposure period (Fig. 5B). The increased activity of GSH-Px may be caused by oxidative stress [49], And the subsequent decline was due to the fact that high temperatures accelerated the denaturation of the enzyme proteins. On the 7th day of aerobic exposure, the T-AOC in all the treatment groups was higher than that in the control group (Fig. 5C); among them, the T-AOCs of the LP + CE group and LP + CE + XY group were the highest, consistent with our previous speculation. In general, the combined LP + enzyme treatment improved the antioxidant activity of *B. papyrifera* silages, which is consistent with the results of previous studies [49].

### Microbial community composition of *B. papyrifera* silages

**Fig. 6:**
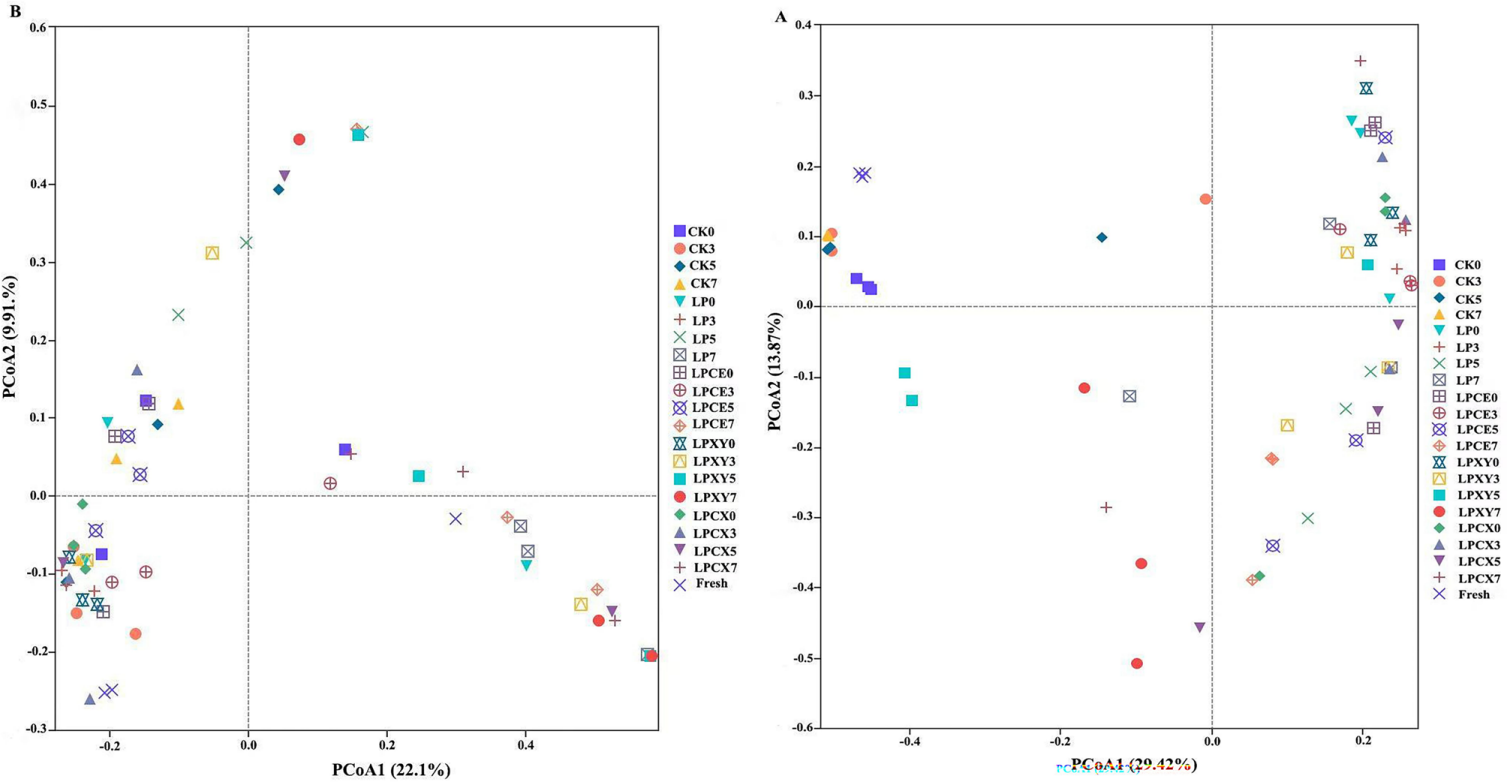
Principal Co-ordinates analysis of (PCoA) bacterial **A** and fungal **B** community composition of *Broussonetia papyrifera* silage fermented with different treatment during the aerobic exposure. Fresh, fresh paper mulberry; LP, *L. plantarum*; LPCE, *L. plantarum* + cellulase; LPXY, *L. plantarum* + xylanase; LPCX, *L. plantarum* + cellulase + xylanase. 0, 0 day; 3, 3 day; 5, 5 day; 7, 7 day

High-throughput sequencing of the 16 S rRNA gene amplicon was performed to systematically characterize the microbial communities in the fresh samples and silage. The α diversity indices (including observed species, Chao1, Simpson and Shannon values) of the silage samples and fresh samples after 0, 3, 5 and 7 days of aerobic exposure are shown in Table 2. In addition to those of the CK group on the third day of aerobic exposure, the bacterial coverage values of all the samples were greater than 0.97, and the fungal coverage values were greater than 0.99, indicating that the depth of the sequencing results reflected the true conditions of the samples well. As shown in the table, the bacterial Os, Chao1, Simpson and Shannon values of the treatment groups on the 3rd, 5th and 7th days were all lower than those of the CK group. The Simpson values of bacteria and fungi on days 5 and 7 were both lower than those in the CK group. These results indicated that the bacterial and fungal diversity decreased after LP, CE and XY supplementation and that the anaerobic environment and low pH prevented most microbes from multiplying [29, 50]. As shown in Fig. 6, the PCoA map reveals the dynamic differences in the bacterial and fungal communities of *B. papyrifera* before and after silage and during the aerobic exposure phase. The bacterial community (PCoA1) and fungal community (PCoA2) accounted for 29.42% and 13.87% of the variance, respectively. Almost all CK and fresh samples were distributed in the second quadrant, suggesting that, in the absence of added processing, the fermentation quality was not different. As shown in Fig. 6A, the added silage samples were well separated from the fresh samples, indicating that their microbial communities were different, consistent with previous research [51]. In Fig. 6B, the LP + CE group was distributed in the second and fourth quadrants, whereas the LP + CE + XY group was distributed in the third quadrant. On the 7th day of aerobic exposure, the fungal communities in the treatment groups were significantly different from those in the control group and were mainly located in the fourth quadrant, indicating the presence of a unique fungal community on the seventh day that was different from that in the early stage of aerobic exposure.

**Table 2:** Alpha diversity of bacteria and fungi in *Broussonetia papyrifera* silage during aerobic exposure

**Items**		**Bacteria**					**Fungi**					**Bacteria/Fungi**	
		**Richness estimator**		**Diversity index**		Coverage	**Richness estimator**		**Diversity index**		Coverage	OS Ratio	Shannon Ratio
Days	Sample	OS	Chao1	Simpson	Shannon		OS	Chao1	Simpson	Shannon			
	Fresh	133.00b	176.87c	0.59e	2.76bc	0.993a	414.67abc	511.14ab	0.81abc	5.27abc	0.998c	2.64b	0.78b
0	CK	60.67b	116.95c	0.56e	1.59c	0.995a	186.67bcde	204.08bcd	0.95a	5.38abc	1.000abc	0.54b	0.3b
	LP	434.67b	586.72bc	0.84abcd	4.46b	0.975ab	231.67bcde	244.41bcd	0.84abc	4.82abc	1.000abc	12.98b	1.31b
	LP+CE	204.00b	262.68c	0.79cd	3.26bc	0.990a	357.33abcd	397.44abcd	0.96a	6.11ab	0.999abc	0.81b	1.41b
	LP+XY	283.67b	355.70c	0.76cd	3.45bc	0.985ab	605.00a	659.17a	0.97a	7.01a	0.999abc	0.52b	0.78b
	LP+CE+XY	251.00b	322.35c	0.81cd	3.61bc	0.987a	385.00abc	425.82abc	0.88abc	5.62abc	0.999abc	0.64b	1.33b
3	CK	976.67a	1497.64a	0.97a	7.65a	0.948c	473.33abc	555.96ab	0.91abc	5.8abc	0.998c	1.80b	0.54b
	LP	278.00b	341.36c	0.79cd	3.49bc	0.986a	521.67ab	561.61ab	0.97a	6.43a	0.999abc	0.54b	29.26b
	LP+CE	182.33b	234.57c	0.81bc	3.29bc	0.991a	295.00abcde	342.78abcd	0.52bcde	2.98cd	0.999abc	1.62b	1666.31a
	LP+XY	248.00b	302.81c	0.88abcd	4.31b	0.990a	145.00cde	212.17bcd	0.39defg	1.68d	0.999abc	9.79b	0.54b
	LP+CE+XY	237.33b	289.63c	0.8cd	3.53bc	0.989a	420.67abc	489.76ab	0.97a	6.24a	0.998abc	0.63b	13.76b
5	CK	890.33a	1035.22ab	0.97ab	7.41a	0.962bc	225.67bcde	239.78bcd	0.93ab	5.19abc	1.000abc	4.99b	1.81b
	LP	289.67b	342.21c	0.88abcd	4.47b	0.989a	45.33de	50.83cd	0.10fg	0.49d	1.000ab	11.78b	62.41b
	LP+CE	206.00b	266.54c	0.81bcd	3.49bc	0.989a	227.00bcde	324.55abcd	0.51cdef	3.11bcd	0.999abc	0.95b	0.64b
	LP+XY	188.00b	267.63c	0.76cd	3.22bc	0.989a	11.67e	11.83d	0.20defg	0.64d	1.000a	19.16b	0.69b
	LP+CE+XY	182.00b	220.75c	0.80cd	3.33bc	0.992a	172.00cde	179.00bcd	0.56abcd	2.79cd	1.000ab	11.26	12.57b
7	CK	194.00b	230.18c	0.92abc	4.78b	0.993a	286.33abcde	291.45abcd	0.96a	6.19a	1.000ab	0.89b	80.12b
	LP	341.33b	433.88bc	0.89abcd	4.72b	0.983ab	11.00e	11.33d	0.11efg	0.47d	1.00a	65.49a	0.52b
	LP+CE	368.33b	521.24bc	0.91abcd	4.70b	0.977ab	7.00e	7.50d	0.04g	0.16d	1.000a	57.23a	3.78b
	LP+XY	359.67b	472.33bc	0.88abcd	4.86b	0.983ab	11.67e	11.67d	0.08g	0.35d	1.000a	22.56b	31.7b
	LP+CE+XY	219.00b	294.4c	0.75d	3.33bc	0.988a	16.00e	22.00d	0.08g	0.30d	1.000a	16.43b	12.08b
SEM		37.40	53.91	0.02	0.21	0.002	29.07	33.13	0.05	0.35	0.0001	3.07	79.03
*P*-value		<0.01	<0.05	<0.001	<0.001	<0.05	<0.001	<0.001	<0.001	<0.001	0.052	<0.05	0.487

Different lowercase letters indicate significant differences of the same column (*P <* 0.05)

*LP* *Lactiplantibacillus plantarum,* *LP + CE* *Lactiplantibacillus plantarum* + cellulase, *LP + XY* *Lactiplantibacillus plantarum* + xylanase, *LP + CE + XY* *Lactiplantibacillus plantarum* + cellulase + xylanase, *OS* Observed species, *SEM* Standard Error of Mean

**Fig. 7:**
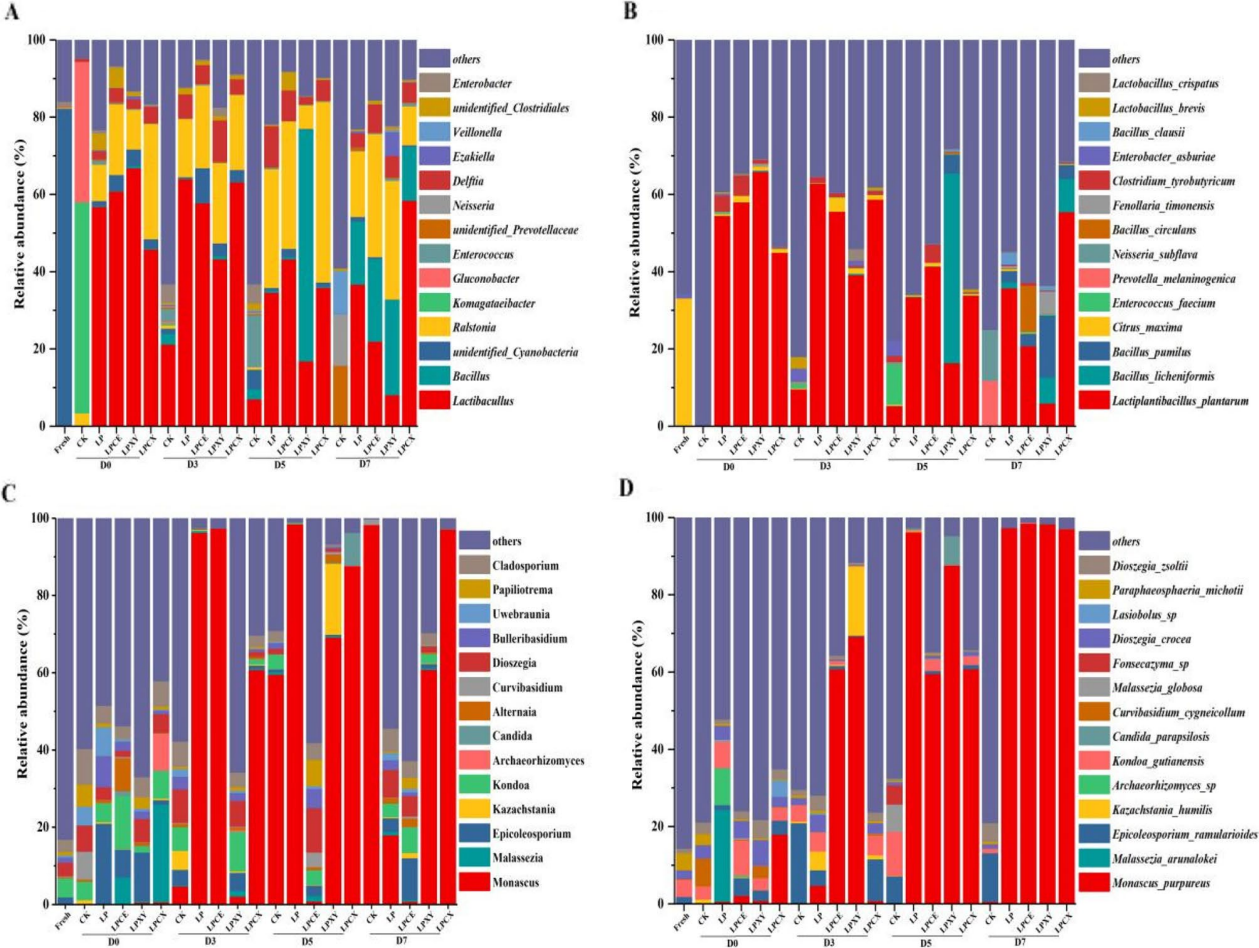
Relative abundance of bacterial and fungal community composition at the genus level **A** and **C** and the species level **B** and **D** of *Broussonetia papyrifera* silage fermented with different treatment during the aerobic exposure. LP, *L. plantarum*; LPCE, *L. plantarum* + cellulase; LPXY, *L. plantarum* + xylanase; LPCX, *L. plantarum* + cellulase + xylanase. D0, 0 day; D3, 3 day; D5, 5 day; D7, 7 day

**Fig. 8:**
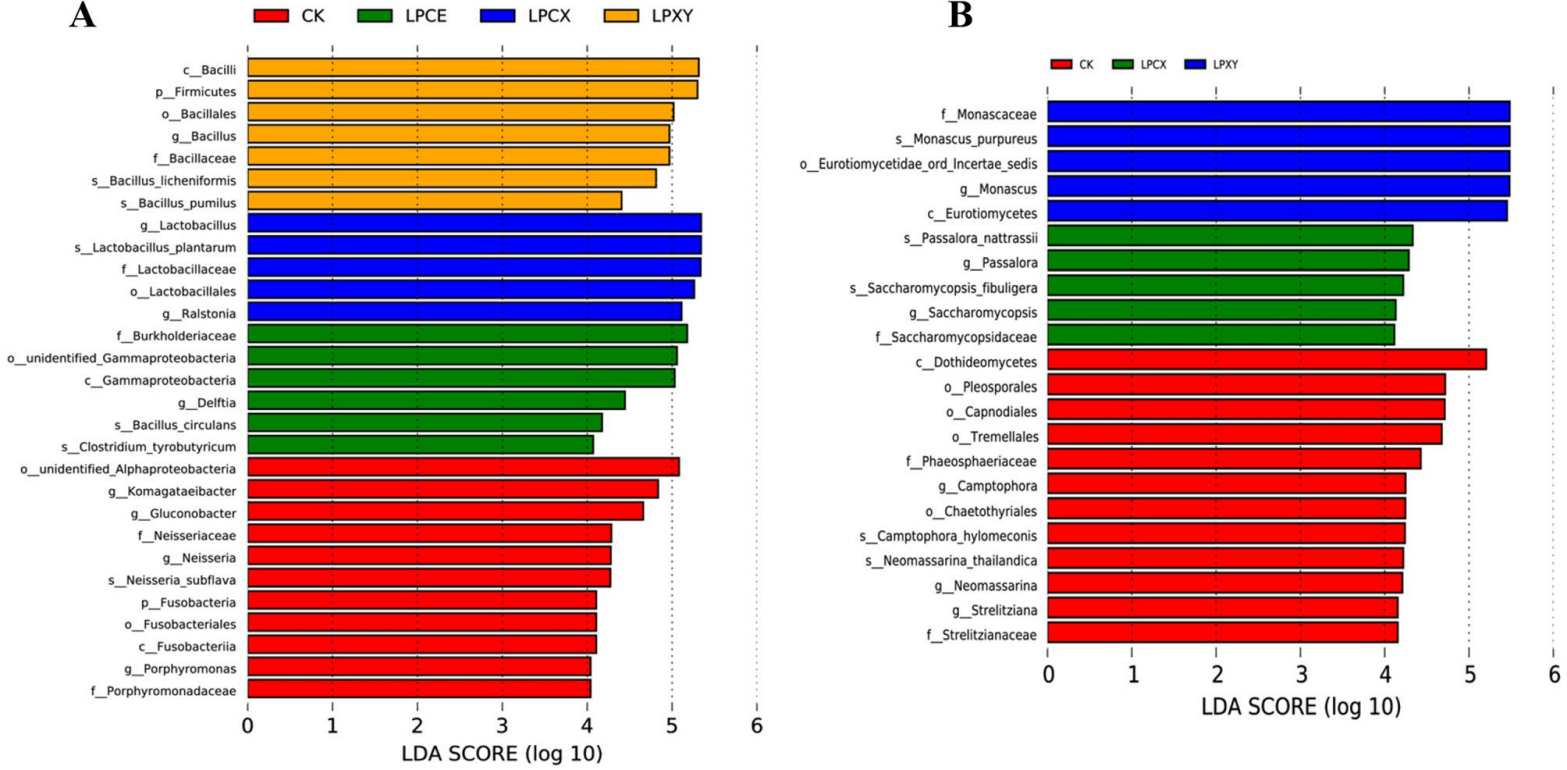
Linear discriminant analysis effect size (LEfSe) of bacterial **A** and fungal **B** community of *Broussonetia papyrifera* silage during the aerobic exposure. LP, *L. plantarum*; LPCE, *L. plantarum* + cellulase; LPXY, *L. plantarum* + xylanase; LPCX, *L. plantarum* + cellulase + xylanase. Identifed taxonomy was signifcantly diferent based on LDA score larger than 4.0

The relative abundances of bacteria at the genus and species levels in the silage and fresh samples are shown in Fig. 7A and B. After 60 days of fermentation, the dominant bacterium in the treatment group was *Lactiplantibacillus*, consistent with the findings reported by Zheng, et al. [52]. The results revealed that silage combined with LP, CE and XY pretreatments effectively inhibited the propagation of spoilage bacteria, increased the production of lactic acid, improved the nutritional value of the feed and prolonged the preservation time. After 60 days of fermentation, the dominant genus in the CK group was *Komagataeibacter*, which is a cellulose-producing strain [53]. The abundance of *Komagataeibacte*r in the treatment group was very low, indicating that the addition of LP significantly decomposed cellulose. During the aerobic exposure process, the relative abundance of *Delftia* in the treatment groups was higher than that in the CK group. *Delftia* is a nonfermentative chemoorganotrophic bacterium that degrades phenolic compounds and anilines in contaminated soil and water [54]. The relatively low pH of silage may be related to the presence of *Delftia* [55]. *Enterobacter* converts LA to AA or other products during ensilage, which results in a degraded silage quality [56]. The relative abundance of *Enterobacter* increased at 0–5 days of aerobic exposure in the CK group, whereas *Enterobacter* was virtually absent in the treatment group. These findings indicate that the addition of LP is effective in inhibiting the propagation of harmful bacteria. As shown in Fig. 7C and D, after 60 days of silage, the abundances of *Cladosporium* and *Paraphaeosphaeria michotii* in the treatment groups were lower than those in the CK group, indicating that the combined treatment could reduce the feed mildew rate [57]. The linear discriminant analysis effect sizes of the bacterial and fungal communities in the different treatment groups during aerobic exposure are shown in Fig. 8A and B. Many unique aerobic bacteria and plant pathogenic fungi were detected in the CK group (e.g., *Neisseria* and *Dothideomycetes)*, which can harm the health of the animals consuming the feed and accelerate the spoilage of silage. In summary, the number of harmful bacteria and fungi significantly affected by differences in the CK group was greater than that in the other treatment groups.

**Fig. 9:**
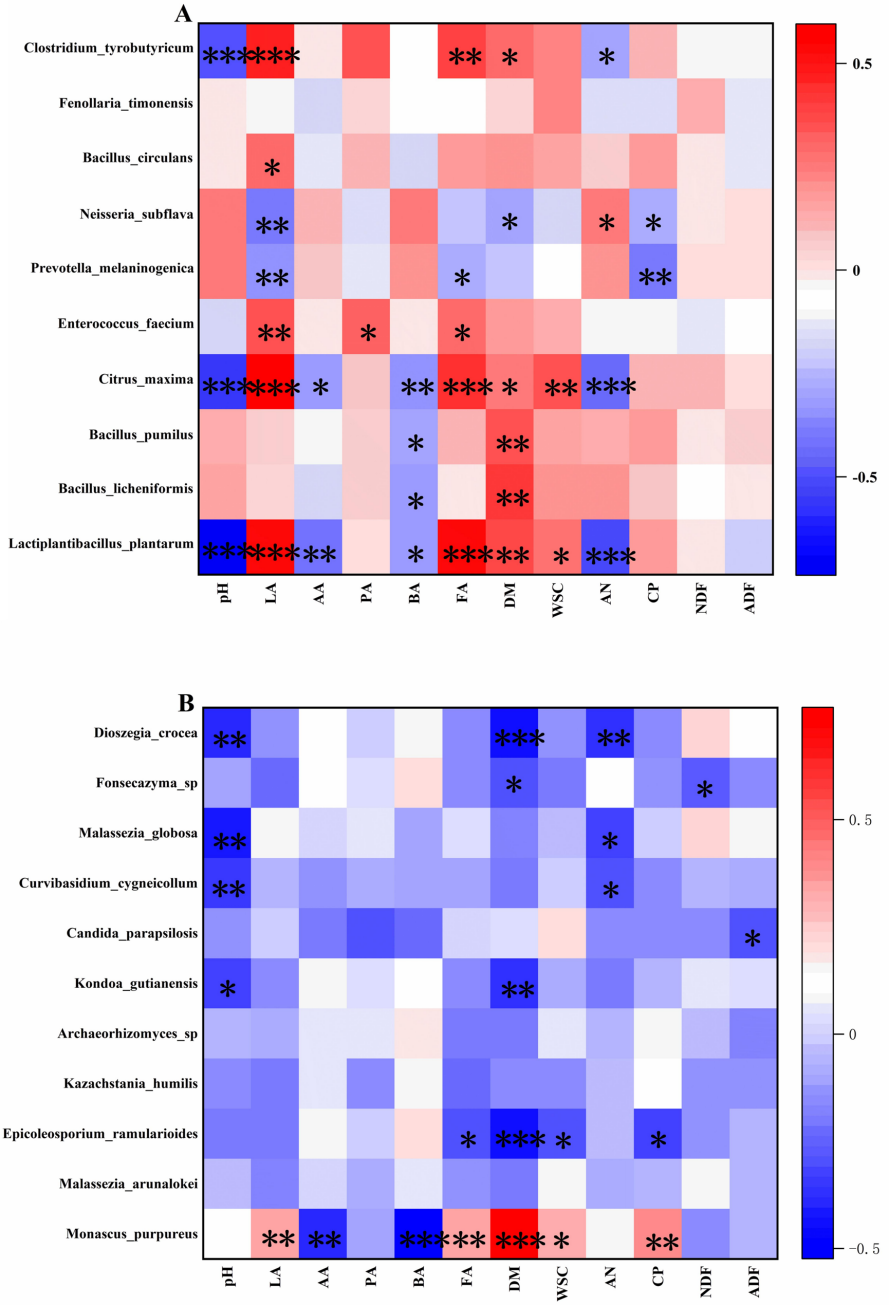
Heatmap of Spearman correlation between silage fermentation parameters and bacterial **A** and fungal **B** community for *Broussonetia papyrifera* silage during the aerobic exposure. LA, lactic acid; AA, acetic acid; PA, propionic acid; BA, butyric acid; FA, ferulic acid; DM, dry matter; WSC, water-soluble carbohydrates; AN, ammonia nitrogen; CP, crude protein; NDF, neutral detergent fibre; ADF, acid detergent fibre. The colours in the heatmaps indicate the Spearman correlation coefficient r, which ranges from − 0.8 to 0.8. *r* < 0 indicates a negative correlation, and *r* > 0 indicates a positive correlation. Asterisks indicate *P < 0.05, **P < 0.01 and ***P < 0.001

Heatmaps of Spearman’s correlation coefficients (Fig. 9) were generated to explore the associations between microbial taxa and silage parameters. *Clostridium tyrobutyricum*, a butyric acid producer, uses lactic acid and acetic acid as substrates [58]. Therefore, the relative abundance of *Clostridium tyrobutyricum* was negatively correlated with the silage pH (*P* < 0.001) and positively correlated with the lactic acid content (*P* < 0.001). The presence of *Lactiplantibacillus plantarum* could reduce the production of NH_3_-N, and thus the relative abundance of *L. plantarum* was negatively correlated with the concentration of ammonia nitrogen (*P* < 0.001). In addition, the ferulic acid concentration was positively correlated with *L. plantarum* (*P* < 0.001) because the addition of *L. plantarum*, which produces ferulate esterase, can degrade the ferulic acid ester bonds and produce FA, and the degradation rate of lignocellulose increases. A greater amount of fermentation substrate is provided for LAB, creating a suitable growth environment for *L. plantarum* and increasing its content and lactic acid concentration (significant positive correlation, *P* < 0.001). *Enterococcus faecium* is a type of intestinal probiotic with excellent in vitro probiotic properties. It has certain inhibitory effects on common pathogenic bacteria in various livestock and aquaculture systems, which contributes to enhancing the fermentation quality of silage [59], and has a significant positive correlation with LA and FA concentrations.

The relative abundance of *Monascus purpureus* was positively correlated with the FA concentration (*P* > 0.01). *Monascus purpureus* can compete with other harmful microorganisms for nutrients and space during silage fermentation and may produce antibacterial substances that inhibit the growth of harmful bacteria and moulds commonly found in silage [60], thus ensuring the quality and safety of silage. *Epicoleosporium ramularioides* carries multiple plant pathogens and can cause yield losses in important crops, such as barley, sugar beet and strawberry [61], and its relative abundance is negatively correlated with the FA concentration. These results demonstrate that FA effectively increases beneficial bacteria populations and inhibits harmful bacteria.

## Conclusions

The combination of LP, CE and XY effectively reduced the pH value, NH3-N concentration and DM loss rate of silage and increased the concentration of LA. Adding LP and CE (LP + CE and LP + CE + XY) significantly increased the concentration of ferulic acid, created a more favourable environment for lactic acid fermentation, and increased the aerobic stability of *B. papyrifera* silage in multiple aspects. The addition of LP increased the abundance of *L. plantarum* and decreased the abundances of *unidentified Cyanobacteria* and *Cladosporium*. Therefore, adding FAE-producing LAB and cellulase could be a feasible method to improve the quality and aerobic stability of *B. papyrifera* silage.

## Data Availability

The raw sequence data have been deposited in the sequence read archive at the NCBI under accession number PRJNA1248703.

## Abbreviations

ADF: Acid detergent fiber
CP: Crude protein
DM: Dry matter
NDF: Neutral detergent fiber
WSC: Water-soluble carbohydrate
